# Evaluating left atrial strain and left ventricular diastolic strain rate as markers for diastolic dysfunction in patients with mitral annular calcification

**DOI:** 10.1007/s10554-023-03041-3

**Published:** 2024-01-30

**Authors:** Edward W. Chen, Zubair Bashir, Jessica L. Churchill, Phinnara Has, Berthold Klas, Gerard P. Aurigemma, Jonathan Bisaillon, John B. Dickey, Philip Haines

**Affiliations:** 1 Department of Internal Medicine, Yale School of Medicine, New Haven, CT USA; 2 Department of Cardiology, Alpert Medical School of Brown University, Providence, RI USA; 3 Lifespan Biostatistics, Epidemiology and Research Design, Rhode Island Hospital, Providence, RI USA; 4 Philips Healthcare, Andover, MA USA; 5 Division of Cardiovascular Medicine, Department of Medicine, University of Massachusetts Chan Medical School, 55 Lake Ave North, Worcester, MA 01655 USA

**Keywords:** Diastolic dysfunction, Mitral annular calcification, Left atrial strain, Left ventricular strain rate, Echocardiography

## Abstract

**Background:**

Mitral annular calcification (MAC) poses many challenges to the evaluation of diastolic function using standard echocardiography. Left atrial (LA) strain and left ventricular early diastolic strain rate (DSr) measured by speckle-tracking echocardiography (STE) are emerging techniques in the noninvasive evaluation of diastolic function. We aim to evaluate the utility of LA strain and early DSr in predicting elevated left ventricular filling pressures (LVFP) in patients with MAC and compare their effectiveness to ratio of mitral inflow velocity in early and late diastole (E/A).

**Methods:**

We included adult patients with MAC who presented between January 1 and December 31, 2014 and received a transthoracic echocardiogram (TTE) and cardiac catheterization with measurement of LVFP within a 24-h period. We used Spearman’s rank correlation coefficient to assess associations of LA reservoir strain and average early DSr with LVFP. Receiver operating characteristic (ROC) curves were computed to assess the effectiveness of LA strain and DSr in discriminating elevated LVFP as a dichotomized variable and to compare their effectiveness with E/A ratio categorized according to grade of diastolic dysfunction.

**Results:**

Fifty-five patients were included. LA reservoir strain demonstrated poor correlation with LVFP (Spearman’s rho = 0.03, p = 0.81) and poor discriminatory ability for detecting elevated LVFP (AUC = 0.54, 95% CI 0.38–0.69). Categorical E/A ratio alone also demonstrated poor discriminatory ability (AUC = 0.53, 95% CI 0.39–0.67), and addition of LA reservoir strain did not significantly improve effectiveness (AUC = 0.58, 95% CI 0.42–0.74, p = 0.56). Average early DSr also demonstrated poor correlation with LVFP (Spearman’s rho = −0.19, p = 0.16) and poor discriminatory ability for detecting elevated LVFP (AUC = 0.59, 95% CI 0.44–0.75). Addition of average early DSr to categorical E/A ratio failed to improve effectiveness (AUC = 0.62, 95% CI 0.46–0.77 vs. AUC = 0.54, 95% CI 0.39–0.69, p = 0.38).

**Conclusions:**

In our sample, LA reservoir strain and DSr do not accurately predict diastolic filling pressure. Further research is required before LA strain and early DSr can be routinely used in clinical practice to assess filling pressure in patients with MAC.

**Supplementary Information:**

The online version contains supplementary material available at 10.1007/s10554-023-03041-3.

## Introduction

Echocardiography plays a key role in the noninvasive evaluation of diastolic function. Multiple parameters are routinely measured in clinical practice to estimate left ventricular filling pressures (LVFP), including mitral valve inflow velocities, mitral annular tissue velocities, and left atrial volume index (LAVI). However, there are many clinical scenarios where these standard 2D echocardiographic parameters do not accurately interpret diastolic function, such as atrial fibrillation or significant tachycardia, significant mitral valve regurgitation or stenosis [1], prosthetic mitral valves, left ventricular assist devices, transplanted hearts, and mitral annular calcification (MAC) [2].

**Fig. 1:**
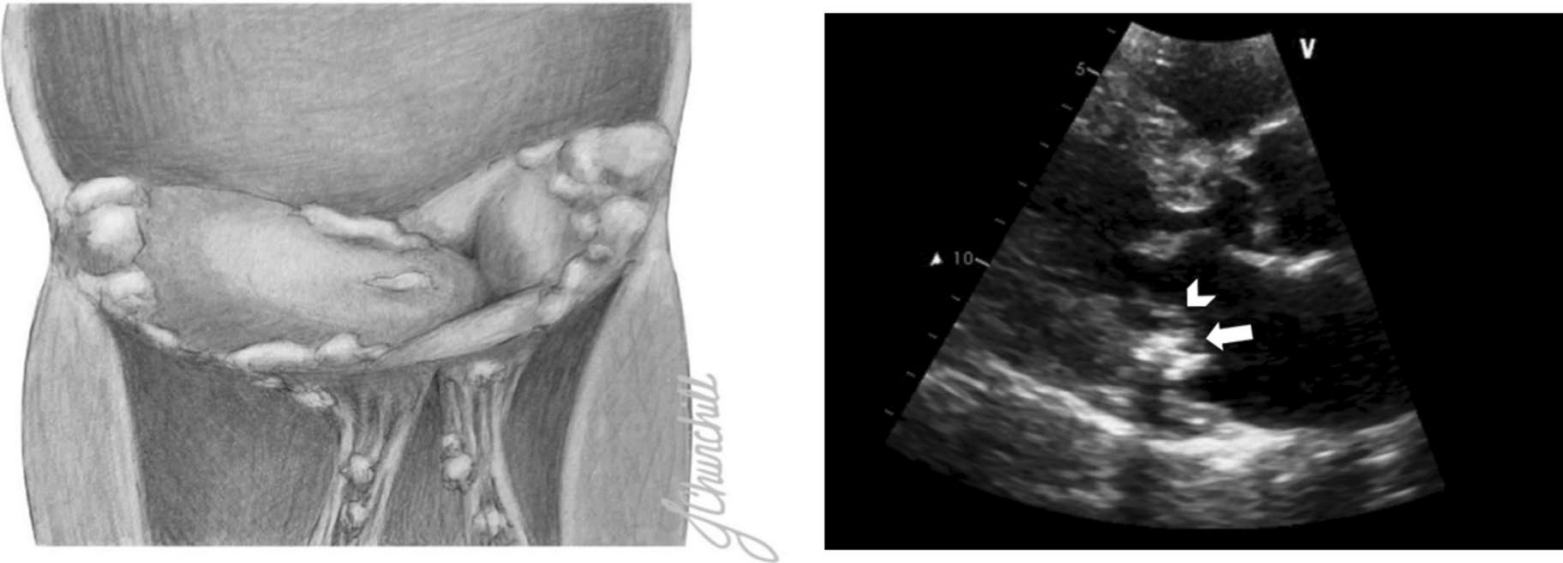
Mitral annular calcification. Illustration (Left Panel) and echocardiogram from the parasternal long axis view of mitral annular calcification. MAC is the progressive deposition of calcium on the fibrous structure of the mitral valve. Calcium typically deposits along the C-shaped ring of the posterior mitral annulus (Right panel, arrow) and base of the posterior leaflet (arrowhead). Calcification typically avoids the anterior annulus but can be present in severe disease. The commissures and leaflet tips are typically spared in MAC, in contrast to rheumatic mitral valve disease

The presence of MAC poses several challenges to the evaluation of diastolic function using standard echocardiographic parameters. Tissue Doppler metrics developed to estimate diastolic filling pressure were derived using normal myocardium and mitral annuli, not calcium. Furthermore, annular calcium may restrict annular motion and alter orifice diameter, resulting in inaccurate spectral Doppler profiles (Fig. 1) [3–5]. Despite a rising prevalence of patients with MAC [6], methods to assess diastolic function noninvasively in settings of MAC are controversial. Abudiab et al. created a clinical algorithm utilizing E/A ratio in combination with isovolumetric relaxation (IVR) time to estimate LVFP in patients with MAC [7]; however, this algorithm failed to demonstrate similar accuracy, sensitivity, and specificity in a recent single center study [8].

Left atrial (LA) strain measured by speckle-tracking echocardiography (STE) is an emerging technique in the noninvasive evaluation of diastolic function [9]. LA strain has been shown to add incremental value to the other standard indices of diastolic function, and in some cases provide a superior assessment to E/eʹ ratio [10, 11] and LAVI [12]. Prior studies suggest that LA strain correlates well with LVFP in patients with early asymptomatic diastolic dysfunction (DD) [13] as well as DD with preserved ejection fraction [10, 14, 15]. LA strain may also serve as a significant marker and prognostic factor in patients with heart failure with preserved ejection fraction (HFpEF) [16–18] and reduced ejection fraction (HFrEF) [19, 20]. Left ventricular (LV) diastolic strain rate (DSr) measured by STE also shows promise as a potential accurate predictor of LVFP [21–23]. Moreover, early DSr offers a comprehensive assessment of global diastolic function, addressing limitations such as angle dependence, sample location, and mitral annular structural pathology [24]. When coupled with early diastolic transmitral inflow velocity (E), the E/DSr ratio can estimate early left ventricular filling pressure [22, 25], serving as a marker for detecting subclinical cardiac disease in patients with preserved ejection fraction and those with fulminant cardiac disease [26, 27]. Additionally, E/DSr has demonstrated prognostic value across various cardiovascular disease populations [28–30]. Both LA strain [31] and LV DSr [32] have been shown to be less load-dependent than traditional echocardiographic parameters.

Thus, our goal is to investigate whether LA strain and LV DSr can accurately predict elevated LVFP in patients with MAC, and how these STE parameters compare to current clinical schema, specifically E/A ratio. We hypothesize that strong correlations exist between reduced LA strain, reduced LV DSr, and elevated LVFP, and that these STE parameters are significantly more accurate than E/A ratio in predicting elevated LVFP.

## Methods

The study population included consecutive patients over 18 years old with MAC who presented to the medical center between January 1st and December 31st, 2014 and received a complete transthoracic echocardiogram (TTE) and a cardiac catheterization with measurement of LVFP within a 24-h period. Patients with atrial fibrillation, prosthetic mitral valves, mechanical support devices (e.g., percutaneous LV assist device), and uninterpretable echocardiograms or LV pressure tracings were excluded. Patient demographic data and vascular risk factors (i.e., hypertension, diabetes, hyperlipidemia, coronary artery disease (CAD), and acute coronary syndrome) were also extracted.

### Echocardiographic parameters

TTE studies were performed with ultrasound systems capable of harmonic and tissue Doppler imaging (GE Medical Systems; Phillips Healthcare) according to guidelines from the American Society of Echocardiography [2, 33]. These studies were transmitted to EchoPAC (GE Medical Systems), on which all Doppler measurements were performed. To acquire peak mitral valve inflow velocity of early (E-wave) and late (A-wave) diastolic filling, pulsed-wave Doppler imaging was performed with the sample volume at the mitral leaflet tips; these measurements were averaged over three cardiac cycles. Assessment of MAC in the posterior annulus was made perpendicular to the long axis of the LV [34]. The phasic LA strain was quantified from the apical four chamber view (A4C) using the 2-D cardiac performance analysis package of TOMTEC software (Chicago, IL) [35], following the guidelines established by the European Association of Cardiovascular Imaging/American Society of Echocardiography Task Force [36]. The software package automatically detected and tracked the endocardial border of the LA which was reviewed and approved. The average global longitudinal LA strain curve generated two peaks consistent with reservoir and contractile strains. The first curve, the peak atrial longitudinal strain (PALS), was calculated as a measure of LA reservoir function (strain) from the atrial strain curve at the end of ventricular systole [36].

**Fig. 2:**
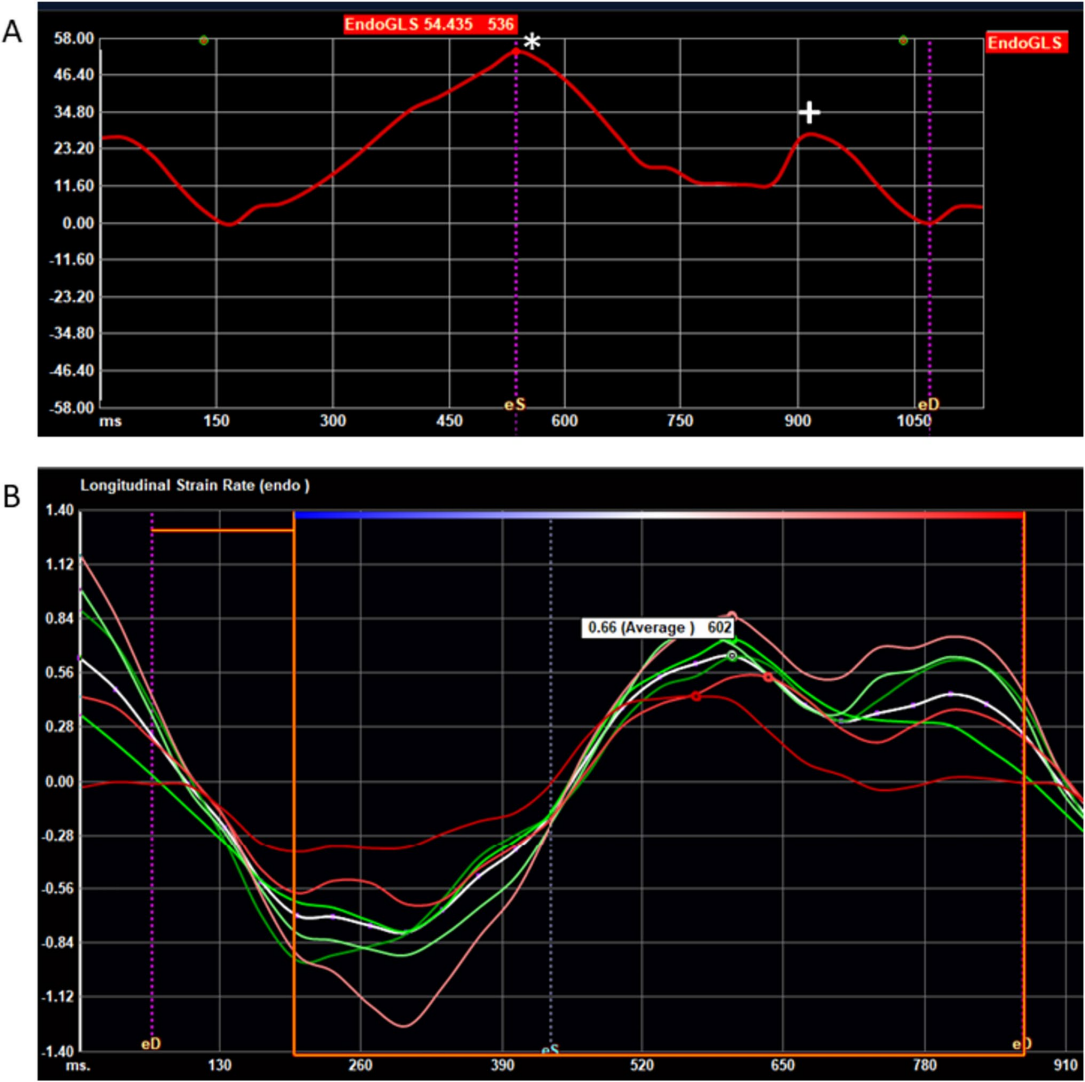
Left atrial strain and left ventricular strain rate curves. **A** Left atrial strain curve. Vertical dashed lines indicate end systole (eS) and end diastole (eD). The first peak (*) marks peak left atrial longitudinal strain (reservoir strain). The second peak (+) marks the peak atrial contractile strain. **B** Left ventricular strain rate curves. Vertical dashed lines indicate end diastole (eD) and end systole (eS). Individual colored lines represent segmental LV analysis of strain rate, with the white line plotting the average of these segments, with peak early diastolic strain rate labeled

Average early DSr was measured using three separate views of the left ventricle: apical four-chamber (A4C), apical two-chamber (A2C), and apical three-chamber (A3C) views. The software package automatically detected endocardial borders and tracked motion throughout the cardiac cycle and divided the LV into basal, mid, and apical segments. Tracking of each segment was reviewed and approved. Peak global early DSr was recorded and averaged for the three views and used for final analysis [21, 37, 38] (Fig. 2).

### Cardiac catheterization parameters

Invasive hemodynamic measurements were performed offline (Mac-Lab, GE Medical systems) and derived from data obtained during the left heart catheterization prior to ventriculography, if performed. LV diastolic pressures before atrial contraction were recorded and averaged over three consecutive cardiac cycles.

### Analyses

Continuous variables were described using means with standard deviation. Categorical variables were described as frequencies and percentage of included patients.

We used Spearman’s rank correlation coefficient to assess the associations of LA reservoir strain and average early DSr with pre-atrial contraction (pre-A) LV pressure as a continuous variable. Receiver operating characteristic (ROC) curves were constructed to assess the abilities of LA reservoir strain and average early DSr to discriminate LVFP as a dichotomized variable (categorized as normal <12 mmHg or elevated ≥12 mmHg) and to discern the optimal cutoff yielding maximum sensitivity and specificity.

The E/A ratio variable was categorized into three groups according to findings from a recent study that maximized the sensitivity and specificity of E/A ratio in detecting elevated LVFP [7]: <0.8, 0.80–1.8, and >1.8. We compared the area under the ROC curves (AUC) of Categorical E/A ratio alone versus Categorical E/A ratio combined with LA reservoir strain or average early DSr to assess whether the addition of either LA reservoir strain or average early DSr to E/A ratio improved discriminatory ability.

Sensitivity analyses using patients who received a TTE and cardiac catheterization with measurement of LVFP within an 8-h duration were conducted to account for any potential acute changes in diastolic filling pressures between time of TTE and catheterization.

A two-sided *p*-value < 0.05 was considered statistically significant. All analyses were performed using Stata/MP16.1 (College Station, TX).

## Results

### Baseline characteristics

**Fig. 3:**
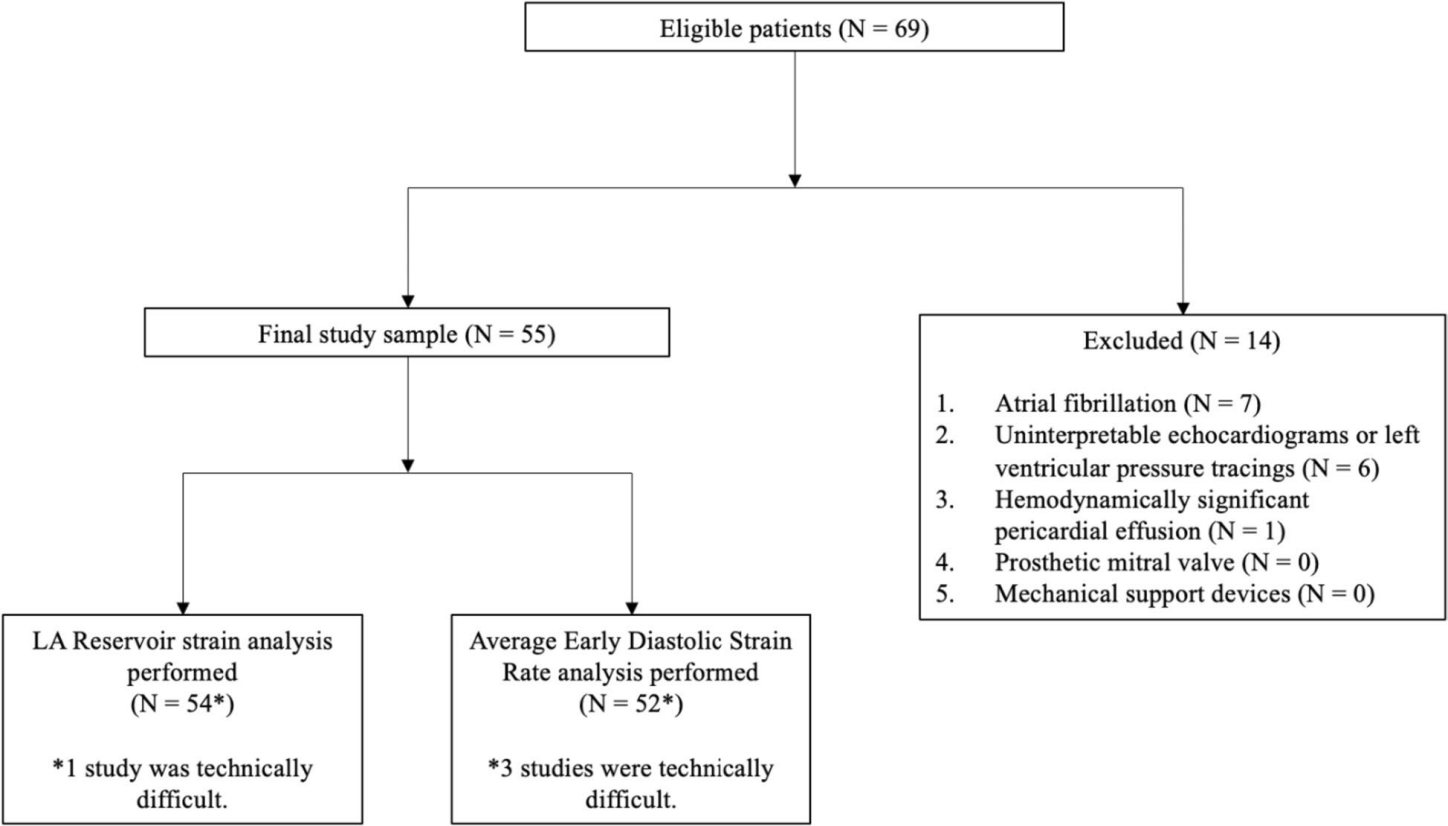
Study population flowchart

Among the 69 eligible patients who had MAC and received both a TTE and cardiac catheterization within a 24-h period, 14 patients were excluded (7 for atrial fibrillation, 6 had technically difficult studies, 1 due to hemodynamically significant pericardial effusion). The final study population comprised 55 patients (Fig. 3). Among these patients, 23 patients received a TTE and cardiac catheterization within 8 h of each other. Mean age was (68.8 ± 11.5 years), and most patients were male (56.4%). Patients were predominately referred for cardiac catheterization for CAD (98.2%) including acute coronary syndrome (56.4%) and had considerable prevalence of hypertension (92.7%), diabetes (38.2%), and hyperlipidemia (92.7%). Mean LA reservoir strain was 23.6 ± 8.58. Mean average early DSr was 0.52 ± 0.21 (Table 1).

**Table 1:** Baseline characteristics and STE measurements of sample population

Variable	No. of patients	Median (IQR) or Frequency (%)
*Demographics*		
Age	55	71 (62–78)
Male	55	31 (56.4%)
Prevalence of hypertension	55	51 (92.7%)
Prevalence of diabetes	55	21 (38.2%)
Prevalence of hyperlipidemia	55	51 (92.7%)
Prevalence of coronary artery disease	55	54 (98.2%)
Prevalence of acute coronary syndrome	55	31 (56.4%)
*Echo parameters*		
LA reservoir strain	54	23.9 (18.4–27.3)
Average early diastolic strain rate	52	0.53 (0.36–0.63)
E/A ratio		
Continuous:		1.06 (0.77–1.45)
Categorical:		
0.8 0.8–1.8 1.8	55	18 (32.3%) 29 (52.7%) 8 (14.6%)
Pre ALVP	55	13 (9–19)

### Comparing LA strain to actual recorded patient LVFP and E/A ratio

**Fig. 4:**
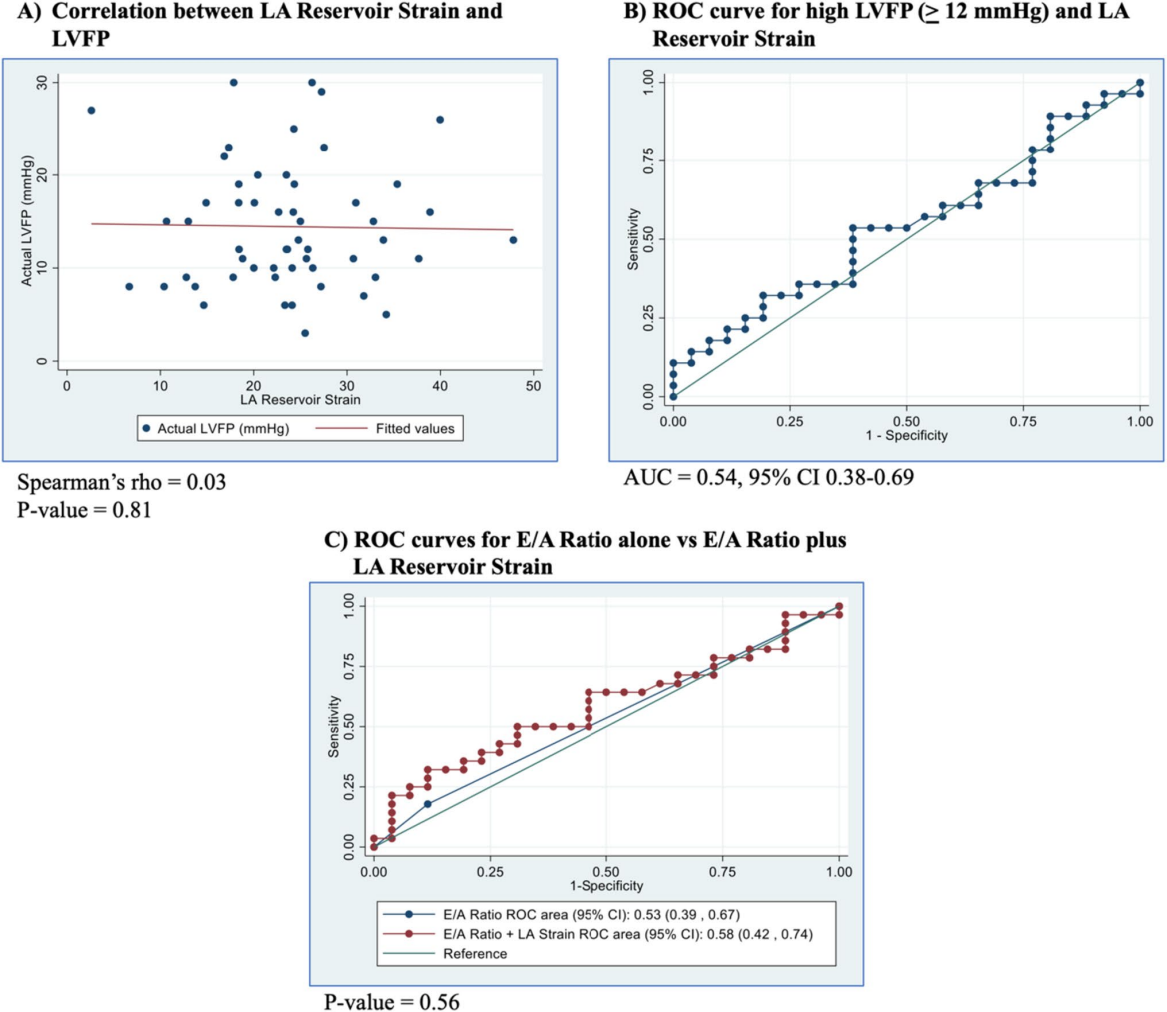
Left atrial reservoir strain analysis

LA reservoir strain showed poor correlation with pre-A LVFP (Spearman’s rho = 0.03, p = 0.81) (Fig. 4A). ROC curve analysis for LA reservoir strain demonstrated poor discriminatory ability for detecting elevated LVFP (AUC = 0.54, 95% CI 0.38–0.69) (Fig. 4B). The optimal cutoff value was 24.24 (sensitivity 53.6%, specificity 61.5%). ROC curve analysis for E/A ratio alone also showed poor discriminatory ability for detecting elevated LVFP (AUC = 0.53, 95% CI 0.39–0.67), and the addition of LA reservoir strain as a continuous variable showed no significant difference in discriminatory ability (AUC = 0.58, 95% CI 0.42–0.74, p = 0.56) (Fig. 4C). Sensitivity analyses using patients who received a TTE and a cardiac catheterization within an 8-h duration yielded similar results (Supplementary Fig. 1).

### Comparing average early diastolic strain rate to actual recorded patient LVFP and E/A ratio

**Fig. 5:**
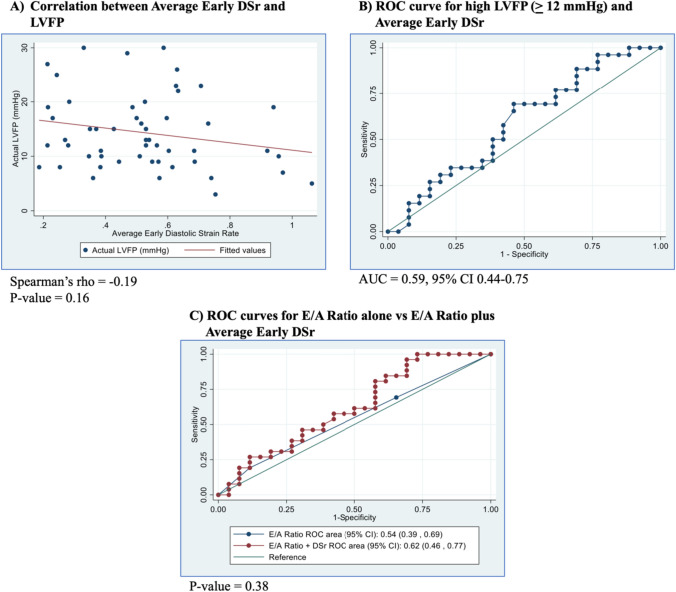
Average early diastolic strain rate analysis

Average early DSr showed poor correlation with pre-A LVFP (Spearman’s rho = −0.19, p = 0.16) (Fig. 5A). ROC curve analysis for average early DSr demonstrated poor discriminatory ability for detecting elevated LVFP (AUC = 0.59, 95% CI 0.44–0.75) (Fig. 5B). The optimal cutoff value was 0.54 (sensitivity 69.2%, specificity 53.9%). Compared to the ROC curve analysis for E/A ratio alone, the addition of average early DSr as a continuous variable showed no significant difference in discriminatory ability (AUC = 0.62, 95% CI 0.46–0.77 vs. AUC = 0.54, 95% CI 0.39–0.69, p = 0.38) (Fig. 5C). Sensitivity analyses using patients who received a TTE and a cardiac catheterization within an 8-h duration yielded similar results, with the exception of average early DSr demonstrating acceptable discriminatory ability for detecting elevated LVFP (AUC = 0.75, 95% CI 0.53–0.97) (Supplementary Fig. 2).

## Discussion

To our knowledge, this is the first study to investigate the utilization of LA reservoir strain and early DSr in patients with MAC. Despite evidence supporting these two STE parameters as markers for diastolic function, our study findings demonstrated poor utility of LA reservoir strain and early DSr in predicting elevated LVFP in patients with MAC. This proved true when LA reservoir strain and early DSr were used as either stand-alone parameters or additions to the current diastolic algorithm. Sensitivity analyses using patients with a shorter TTE-to-catheterization interval of 8 h yielded largely similar results, with the exception of early DSr showing improved ability to detect elevated LVFP.

### Mechanism of association

LA phasic function (reservoir, conduit, and contractile) is integral in modulating LV diastolic filling [39]. Changes in LA function become evident in early stages of diastolic dysfunction before more obvious changes in LA and LV structure and function [40]. Therefore, there has been increased attention in investigating LA strain’s utility as an early marker for diastolic dysfunction. Singh et al. demonstrated LA strain’s ability to accurately differentiate between grades of diastolic dysfunction in patients with HFpEF [14]. Another recent study by Inoue et al. found significant associations of LA reservoir and contractile strains with LVFP in patients with HFrEF [20]. LA strain has also been shown to improve diagnostic accuracy of the 2016 ASE/EACVI diastolic algorithm in detecting elevated LVFP in patients with preserved left ventricular ejection fraction [15].

Wang et al. were the first to demonstrate the utility of DSr in assessing diastolic function, finding that global DSr during the IVR period relates well to LV relaxation and that E/DSr during IVR (E/SRIVR) ratio can accurately predict filling pressures in dogs and patients with normal ejection fractions [21]. Dokainish et al. further expanded on these findings, demonstrating that E/DSr ratio is more accurate than E/eʹ in predicting LVFP in a similar patient population [22]. Hatipoglu et al. confirmed many of these findings and suggested that SRIVR may be a superior measure of diastolic function than E/SRIVR [23].

### Clinical implications and limitations

Assessing LVFP noninvasively in the setting of MAC has been a frustrating problem. The 2016 update to the ASE diastolic function guidelines is silent on recommendations to assess LVFP in this population. Abudiab et al. published a decision tool incorporating isovolumic relaxation time with E/A ratio demonstrating apparently good accuracy for LVFP [7]. However, a subsequent independent assessment of their algorithm failed to replicate their findings [8]. Recent literature highlighting LA strain and DSr as novel methods to assess LVFP, both as independent markers and in combination with traditional echocardiographic diastolic parameters, led us to our hypothesis that they would be useful in a population in MAC. Unfortunately, our results do not confirm this hypothesis, although early DSr had modest discriminatory capability and should be further assessed in future studies.

There are several limitations in our study. First, we acknowledge that the time window between cardiac catheterization and echocardiogram in this retrospective analysis introduces confounders into the relationship between invasive and non-invasive measurements. Several dynamic factors during this time could potentially alter LVFP and weaken the association between pressure measurements taken at one time point and echocardiography performed at another time point. Restricting the time interval to less than 8 h did not improve the performance of LA reservoir strain and only marginally improved performance of early DSr, suggesting that the impact of the time delay may be negligible. Additionally, an animal model of acute ischemic heart disease suggests the relationship between non-invasive filling pressure estimation and invasive hemodynamic measurements remains correlated up to 2 days between assessments, weakening arguments that prolonged periods between echocardiogram and catheterization would uncouple a robust correlation [41]. These findings highlight potential limitations of echocardiography to temporally track acute changes in cardiac hemodynamics, suggesting echocardiography is better at determining subacute or chronic hemodynamic effects.

Second, the vast majority of our patient population had CAD which may limit the generalizability of our findings across the spectrum of MAC patients. However, previous studies have demonstrated good correlation of reduced LA reservoir strain with LVFP in patients with stable CAD [42]. This is likely due to impaired LV myocardial function causing chronically elevated left-sided pressures and subsequently LA volumetric changes and remodeling. Atrial remodeling impairs the LA compliance which manifests as reduced LA reservoir strain. In addition, reduced LA reservoir strain has also shown moderate correlation with sudden elevation in LVFP in ACS patients with myocardial stunning prior to any changes in LA volume [43]. Lower LV early DSr is also significantly associated with elevated LVFP in CAD patients [44]. Tanaka et al. reported improvement in early DSr in ACS patients post percutaneous coronary intervention (PCI) indicating recovery of LV myocardium and improvement in LV relaxation [45]. These findings suggest that elevated LVFP due to impaired LV relaxation is detected by reduced early DSr. This reiterates the sensitivity of LA reservoir strain and early DSr in identifying sudden changes in LVFP. Interestingly, our study with a dominant CAD population revealed poor correlation of LA reservoir strain and early DSr with LVFP. We believe further studies are needed to ascertain the impact of the severity of MAC on predicting elevated LVFP using LA reservoir strain and LV early DSr across the spectrum of patients with MAC, including those with CAD.

We propose the following hypotheses to explain our findings. MAC itself may be independently associated with LA strain or early DSr, thus obscuring any potential relationships between LA strain, early DSr, and LVFP. However, our sample size is not sufficiently large enough to investigate these relationships. MAC may also be associated with other pathologies that influence LA strain and DSr. Indeed, MAC is a chronic, degenerative process associated with increased age, atherosclerosis, and other cardiovascular risk factors [6] that may affect LA and LV function and structure. Lastly, MAC may interfere with ultrasound transmission, potentially producing artifact that obscures the left atrial wall and thus prevents accurate strain analysis.

### Future directions

Our study calls for further research before LA strain and early DSr can be routinely used in clinical practice to assess diastolic function, specifically in patients with MAC. Studies that are prospective in nature, minimize the time between cardiac catheterization and echocardiogram, stratify patients by degree of MAC, and include larger, more diverse sample sizes are necessary to elucidate any relationships between MAC, LA strain, early DSr, and LVFP. Our findings also call for research studying whether different thresholds for E/A ratio, LA strain, and early DSr are necessary for evaluating diastolic function in MAC patients specifically and in other subpopulations where traditional echocardiographic parameters are insufficient.

## Conclusion

Our study is the first to evaluate the utility of LA strain and LV early DSr in assessing diastolic function specifically in patients with MAC. Despite evidence supporting their use in other populations, we found no significant associations between LA strain, LV early DSr, and LVFP in patients with MAC. Furthermore, LA strain and LV early DSr had poor discriminatory ability for detecting elevated LVFP. Addition of these parameters to current clinical schema using E/A ratio did not result in any significant improvement in detecting elevated LVFP. Further studies with larger and more diverse sample sizes are necessary to elucidate the relationship between MAC, LA strain, LV early DSr, and LVFP.

### Supplementary Information

Below is the link to the electronic supplementary material.

Supplementary file1 (DOCX 237 KB)

Supplementary file2 (DOCX 242 KB)

## Data Availability

The data that support the findings of this study are available from the corresponding author upon reasonable request. The data are not publicly available due to privacy or ethical restrictions.

## Abbreviations

MAC: Mitral annular calcification
LV: Left ventricle
LVFP: LV filling pressure
LAVI: Left atrial volume index
STE: Speckle-tracing echocardiography
DSr: Diastolic strain rate
ACS: Acute coronary syndrome
CAD: Coronary artery disease
PCI: Percutaneous coronary intervention
DD: Diastolic dysfunction
