# Supplementary material for: Evaluating left atrial strain and left ventricular diastolic strain rate as markers for diastolic dysfunction in patients with mitral annular calcification
Source: Int J Cardiovasc Imaging. 2024 Jan 30;40(4):733–43. doi: 10.1007/s10554-023-03041-3 (PMC11052839; doi:10.1007/s10554-023-03041-3)
Supplement: Supplementary file 2 — Supplementary file2 (DOCX 242 KB) [file 10554_2023_3041_MOESM2_ESM.docx]

**Supplementary Figure 2. Average Early Diastolic Strain Rate Analysis in Patients with Echo-Cath Interval within 8 hours**

**
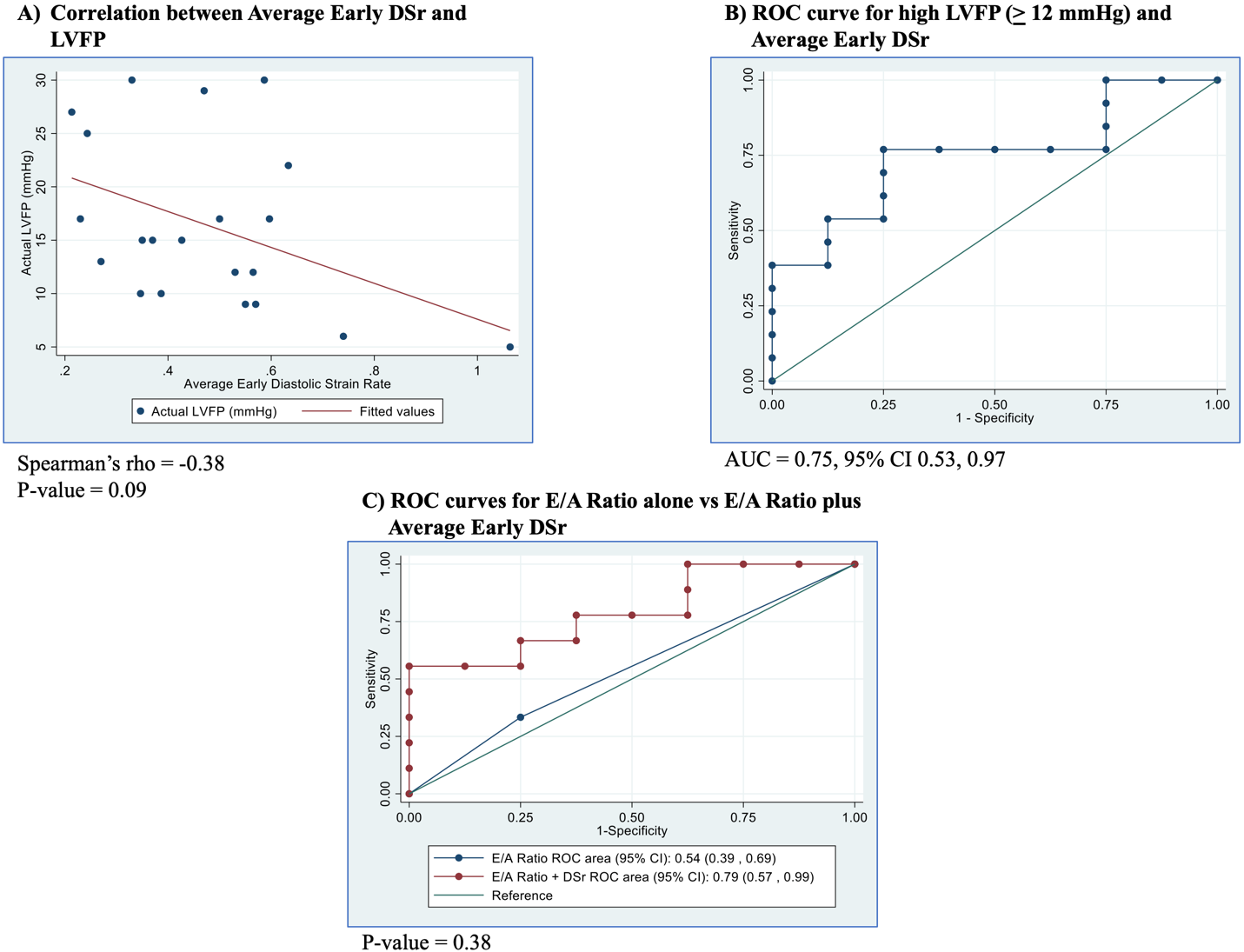
**
